# COST AND QUALITY OF LIFE IN PATIENTS UNDERWENT HIP ARTHROPLASTY IN A BRAZILIAN HOSPITAL

**DOI:** 10.1590/1413-785220253302e280114

**Published:** 2025-06-02

**Authors:** Maria-Roxana Viamont-Guerra, Alex Takao Sasai, Júlia Martins Rodrigues, Rodrigo Guimarães, Eliane Antonioli, Mario Lenza

**Affiliations:** 1 Hospital Israelita Albert Einstein, Sao Paulo, SP, Brazil.

**Keywords:** Arthroplasty, Hip, Quality of life, Hospital costs, Artroplastia, Quadril, Qualidade de Vida, Custos hospitalares

## Abstract

**Introduction:**

There are few studies in Brazil evaluating the quality of life and costs of patients undergoing total hip arthroplasty (THA).

**Purpose:**

(1) To describe the total cost, quality of life and functional scores of patients undergoing THA in a Brazilian private hospital; (2) to determine preoperative factors associated with quality of life and total cost; and (3) to evaluate the association between cost and quality of life.

**Methods:**

Of the 1061 patients included, pre- and peri-operative data, total costs, pre- and post-operative functional scores (HOOS, WOMAC) and quality of life scores (EQ-5D) were collected over 2 years.

**Results:**

EQ-5D, HOOS and WOMAC improved from 0.492, 54.5 and 45.2, respectively, to 0.888, 90 and 5.9 at 1-year follow-up, and 0.892, 90.4 and 4.0 at 2-year follow-up. The average cost was R$43,324.22±11,323. Associations were observed between EQ-5D variation and male sex (RM=1.058; p=0.03) as well as age (RM=0.996; p<0.001). No association was found between total cost and EQ-5D.

**Conclusions:**

The average cost of patients undergoing THA was R$43,324.22. There was an improvement in quality of life and function, an association between quality of life and male sex and age, however no association was found between total cost and quality of life. ***Level of evidence IV; Case Series.***

## RESUMO

**Introdução:**

Há escassez de estudos no Brasil que avaliem qualidade de vida e custos de pacientes submetidos à artroplastia total do quadril (ATQ).

**Objetivos:**

(1) Descrever custo total, escores de qualidade de vida e funcionais de pacientes submetidos à ATQ num hospital privado brasileiro; (2) determinar fatores pré-operatórios associados com qualidade de vida e custo total; e (3) avaliar associação entre custo e qualidade de vida.

**Método:**

Dos 1061 pacientes incluídos, foram coletados dados pré e peri-operatórios, custo total, escores funcionais (HOOS, WOMAC) e de qualidade de vida (EQ-5D) pré- e pós-operatórios ao longo de 2 anos.

**Resultados:**

EQ-5D, HOOS e WOMAC melhoraram de 0,492, 54,5 e 45,2, respectivamente, para 0,888, 90 e 5,9 com 1 ano de seguimento, e 0,892, 90,4 e 4,0 com 2 anos. O custo médio foi R$43.324,22±11.323. Observou-se associações da variação de EQ-5D com sexo masculino (RM=1,058; p=0,03) e idade (RM=0,996; p<0,001). Não observou-se associação entre custo total e EQ-5D.

**Conclusão:**

O custo médio de pacientes submetidos à ATQ foi R$43.324,22. Observou-se melhora da qualidade de vida e função, associação da qualidade de vida com sexo masculino e idade, porém não encontrou-se associação entre custo total e qualidade de vida. ***Nível de evidencia IV; Série de Casos.***

## INTRODUCTION

Total hip arthroplasty (THA) is considered one of the most successful and cost-effective surgeries when compared to other orthopedic surgeries.^1,2^ It is estimated that, with an ageing population, there will be an increase in demand for THAs,^3,4^ and there is concern about the impact on health system costs.

In Australia, based on the national arthroplasty registry, there was an increase in the annual cost of THA surgeries from 2003 to 2013, from 364 million to 625 million Australian dollars ($AUD), with the expectation that by 2030 this cost will rise to $AUD 953 million.^5^ In Brazil, there are few studies on the costs of THA. Recently, Guimarães et al. evaluated the costs of THA in the public health system in the city of São Paulo and found average costs per patient of 1,345.15 and 1,840.42 dollars for cemented and uncemented/hybrid THA, respectively.^6^

Patients undergoing THA, due to arthrosis or other hip deformity pathologies in more advanced stages, evolve with significant improvement in pain, function and quality of life.^7^ Quality of life in the post-THA period can be measured using the *Euroqol-5 Dimensions* (EQ-5D), a questionnaire that assesses the individual's health in five domains: mobility, self-care, daily activities, pain and anxiety/depression.^8,9^ In addition, there are functional questionnaires, such as the *Hip disability and Osteoarthritis Outcome* score (HOOS) and *the Western Ontario and McMaster Universities Arthritis Index* (WOMAC), which assess aspects related to pain and hip function in patients’ daily activities.^10,11^ These tools analyze the patient's perception of quality of life and restoration of function in relation to the treatment received over time. In Brazil, there is a scarcity of studies evaluating costs, associated or not with preoperative characteristics, clinical outcomes and quality of life of patients undergoing elective THA, both in private and public services. Thus, this study aims to (1) describe the quality of life and functional assessment scores over two years and total cost of patients undergoing elective primary THA in a private tertiary hospital in Brazil; (2) determine preoperative factors associated with quality of life and total cost; and (3) evaluate the association between total cost and quality of life.

## METHODS

The present study is a retrospective cohort with data from patients who underwent elective primary THA in a private tertiary hospital in Brazil with an open clinical staff, considered to be one of the reference centers in orthopedics in Latin America. The study included 1061 patients over the age of 18 who underwent THA for osteoarthritis, osteonecrosis, rheumatoid arthritis or hip dysplasia between 2012 and 2019.

The hospital has been systematically collecting and recording data on all patients undergoing THA, such as demographic data, preoperative data, perioperative data, hospitalization data and the need for readmission, since 2009. However, data on clinical and functional outcomes over time began to be collected and recorded in 2012. Cost data is stored in another database in the hospital's administrative department, which can be consulted with institutional authorization.

For this study, the following pre- and peri-operative data was collected: gender, age, body mass index (BMI), comorbidities (hypertension, diabetes, heart disease, smoking), diagnosis of the hip pathology affected, preoperative quality of life using the EQ-5D,^8^ preoperative functional scores using the HOOS^11^ and WOMAC,^10^ surgery time, length of hospital stay, place of hospitalization on the first postoperative day (ward, intensive care unit (ICU), semi-ICU), and need for readmission within 30 days. The total cost of the procedure was also collected, which involves the costs related to the hospitalization period directly related to the surgical procedure, such as hospitalization time, operating room use time, list of medications, surgical materials and implants used during surgery. In addition, postoperative data from the EQ-5D, HOOS and WOMAC scores at 1 and 2 years of follow-up were collected.

This study was approved by the institution's Research Ethics Committee (CAAE: 44554121.1.0000.0071, opinion number 4.704.702) and the informed consent form was waived.

### Statistical analysis

The data was described using absolute and relative frequencies for the qualitative variables and means, standard deviation (SD), minimum and maximum values for the quantitative variables. The distribution of normality was checked using the Shapiro-Wilk test. To compare the questionnaire scores between the different assessment times, generalized mixed models were adjusted and Mann-Whitney parametric tests were used. Comparisons between gender and age group (up to 55 years, between 56 and 70 years and over 70 years) in relation to variations in preoperative and postoperative scores at one and two years of follow-up were assessed using linear mixed models, taking into account the dependence between assessments in the same patient. Associations between the variation in EQ-5D two years after surgery and the total cost with pre- and perioperative data were made using generalized linear models. The correlations between the total cost and the EQ-5D at the end of two years were measured using Spearman's correlation coefficients. The analyses were carried out using the SPSS program, considering a significance level of 5%.

## RESULTS

Of the 1061 patients included, 531 were women (50%) and 530 men (50%) (Table 1). The average age was 62.0 ±14.0 years and the average BMI was 27.6 ±4.5. 72.9% of the patients were overweight or obese. The most frequent comorbidities were hypertension (n=392; 36.9%) and diabetes (n=149; 14.0%). The main diagnosis for the indication of THA was osteoarthritis (96.3%) and the average total cost per patient was R$43,324.22 ±11,323.

**Table 1:** Patients’ pre- and peri-operative data (n=1061).

	n (%)
	Mean ± SD (Min-Max)
Female	531 (50.0%)
Age (years)	62.0 ± 14.0 (18-99)
IMC (kg/m2); n=1025	27.6 ± 4.5 (14.7-43.0)
Low weight (<18.5kg/m²)	8 (0.8%)
Adequate (18.5 to 24.9 kg/m²)	269 (26.2%)
Overweight (25 to 29.9 kg/m²)	435 (42.4%)
Obesity (≥30 kg/m²)	313 (30.5%)
**Comorbidities**	
Hypertension	392 (36.9%)
Diabetes	149 (14.0%)
Heart disease	105 (9.9%)
Smoking	51 (4.8%)
**Diagnosis of hip pathology**	
Osteoarthritis	1022 (96.3%)
Osteonecrosis	33 (3.1%)
Artrite Reumatóide	4 (0.4%)
Hip dysplasia	2 (0.2%)
Surgery time (hours); n = 1020	2.0 ± 0.8 (0.7-8.8)
Length of stay (days)	4.6 ± 3.8 (1.0-76.0)
Readmission within 30 days	3 (0.3%)
**Place of hospitalization in the 1st PO**	
Infirmary	531 (50.0%)
Semi-ICU	178 (16.8%)
ICU	352 (33.2%)
Total cost per patient (reais)	43.324,22 ± 11.323,00 (16.868,31-147.082,01)

SD, standard deviation; n, number of patients; BMI, body mass index; PO, postoperative; ICU, intensive care unit

There was an improvement in all the quality of life and functional assessment scores 1 year after surgery and this improvement was maintained 2 years later (Table 2). At the end of two years, the average EQ-5D improved from 0.492 to 0.892, HOOS improved from 54.5 to 90.4 and WOMAC improved from 45.2 to 4.0.

**Table 2:** Results of quality of life and functional assessment of patients.

	Mean (95% CI)	p-value
**EQ-5D (n=651)**		
Preoperative	0.492 (0.477-0.507)	
Post-surgery 1 year	0.888 (0.872-0.904)	*<0.001
Post-surgery 2 years	0.892 (0.874-0.910)	*<0.001
**HOOS (n=244)**		
Preoperative	54.5 (51.5-57.6)	
Post-surgery 1 year	90.0 (87.7-92.3)	*<0.001
Post-surgery 2 years	90.4 (87.3-93.7)	*<0.001
**WOMAC (n=411)**		
Preoperative	45.2 (42.9-47.6)	
Post-surgery 1 year	5.9 (4.9-7.2)	*<0.001
Post-surgery 2 years	4.0 (3.1-5.1)	*<0.001

* in relation to the preoperative score

Comparisons of score variations showed differences in the EQ-5D between the groups of patients aged up to 55 years and over 70 years in the one-year (p<0.001) and two-year (p<0.001) post-operative evaluations, and between the groups aged 56 to 70 years and over 70 years in the one-year (p=0.003) and two-year (p=0.013) post-operative evaluations (Table 3). There were no differences in the comparisons of the variations in the HOOS and WOMAC scores preoperatively and at one and two years of follow-up between sex and the age groups established.

**Table 3:** Comparisons of variations in EQ-5D, HOOS and WOMAC scores preoperatively and at one and two years of follow-up, according to age group.

	Age		
	Up to 55 years old	56 to 70 years old	Over 70
**EQ-5D scale**			
1 year	0.45 (0.41; 0.49)	0.42 (0.39; 0.45)	0.34 (0.30; 0.38)
2 years	0.47 (0.43; 0.51)	0.42 (0.39; 0.45)	0.35 (0.31; 0.39)
	**Contraste**	**p-value**	
**Evaluation 1 year**			
Up to 55 years old - 56 to 70 years old	0.03 (−0.02; 0.08)	0.305	
Up to 55 years old - over 70 years old	0.11 (0.04; 0.18)	<0.001	
56 to 70 years old - over 70 years old	0.08 (0.02; 0.14)	0.003	
**Evaluation 2 years**			
Up to 55 years old - 56 to 70 years old	0.05 (0.00; 0.10)	0.06	
Up to 55 years old - over 70 years old	0.12 (0.05; 0.19)	<0.001	
56 to 70 years old - over 70 years old	0.07 (0.01; 0.13)	0.013	
**HOOS scale**			
1 year	31.2 (23.9; 38.5)	40.9 (35.1; 46.8)	34.6 (27.0; 42.1)
2 years	34.4 (24.1; 44.8)	39.7 (32.6; 46.8)	35.1 (25.2; 45.0)
	**Contraste**	**p-value**	
**Evaluation 1 year**			
Up to 55 years old - 56 to 70 years old	-9.7 (−21.1; 1.8)	0.127	
Up to 55 years old - over 70 years old	-3.3 (−13.8; 7.2)	0.534	
56 to 70 years old - over 70 years old	6.4 (−4.5; 17.3)	0.378	
**Evaluation 2 years**			
Up to 55 years old - 56 to 70 years old	-5.3 (−20.6; 10.0)	>0.999	
Up to 55 years old - over 70 years old	-0.6 (−15.2; 13.9)	>0.999	
56 to 70 years old - over 70 years old	4.6 (−9.5; 18.8)	>0.999	
**WOMAC scale**			
1 year	−45.5 (−50.1; −40.9)	−40.3 (−44.0; −36.7)	−44.6 (−48.8; −40.4)
2 years	−47.1 (−51.4; −42.8)	−42.6 (−46.0; −39.1)	−45.0 (−48.9; −41.0)
	**Contraste**	**p-value**	
**Evaluation 1 year**			
Up to 55 years old - 56 to 70 years old	−5.1 (−12.3; 2.1)	0.264	
Up to 55 years old - over 70 years old	−0.9 (−7.1; 5.4)	0.785	
56 to 70 years old - over 70 years old	4.2 (−2.1; 10.6)	0.272	
**Evaluation 2 years**			
Up to 55 years old - 56 to 70 years old	−4.5 (−11.3; 2.3)	0.331	
Up to 55 years old - over 70 years old	−2.1 (−8.5; 4.3)	0.745	
56 to 70 years old - over 70 years old	2.4 (−3.6; 8.4)	0.745	

Values expressed as estimated means (95% CI)

The analysis of associations between EQ-5D variation two years after surgery and pre- and peri-operative data showed a significant association with male gender (RM=1.058; p=0.03) and age (RM=0.996; p<0.001) (Table 4).

**Table 4:** Associations of EQ-5D variation two years after surgery with pre- and peri-operative data.

	Estimated means (95% CI)	RM (IC 95%)	p-value
Male (n=156)	0.447 (0.411; 0.483)	1.058 (1.005; 1.113)	0.03
Female (n=162)	0.391 (0.355; 0.426)	1	
Age (years) (n=318)		0.996 (0.995; 0.998)	<0.001
IMC (kg/m2) (n=310)		1.003 (0.997; 1.009)	0.399
Smoker (n=18)	0.440 (0.333; 0.548)	1.024 (0.917; 1.143)	0.676
Non-smoker (n=300)	0.417 (0.391; 0.443)	1	
Hypertensive (n=127)	0.412 (0.372; 0.452)	0.990 (0.940; 1.043)	0.696
Not hypertensive (n=191)	0.422 (0.390; 0.455)	1	
Diabetic (n=53)	0.403 (0.341; 0.466)	0.982 (0.917; 1.052)	0.603
Non-diabetic (n=265)	0.421 (0.393; 0.449)	1	
Heart disease (n=40)	0.384 (0.312; 0.456)	0.961 (0.890; 1.038)	0.314
Non-cardiac (n=278)	0.423 (0.396; 0.450)	1	
Surgery time (hours) (n=303)	1.008 (0.975; 1.042)	0.621	
Length of stay (days) (n=318)	0.991 (0.980; 1.002)	0.097	
1st PO in Infirmary (n=155)	0.432 (0.396; 0.469)	1.019 (0.961; 1.079)	0.531
1st PO in Semi-ICU (n=61)	0.390 (0.332; 0.448)	0.977 (0.908; 1.051)	0.53
1st PO in ICU (n=102)	0.414 (0.369; 0.459)	1	

BMI, body mass index; MR, ratio of means; 95% CI, 95% confidence interval for the estimated ratio of means; reference categories have 1 in place of the odds ratio; PO, postoperative; ICU, intensive care unit.

When analyzing the association between total cost and pre- and perioperative data, a significant association was found with patients who smoked (RM= 0.914; p= 0.043) (Table 5). The other linear analyses showed no evidence of an association between the variation in EQ-5D two years after surgery or the total cost with pre- or peri-operative data.

**Table 5:** Associations between total cost and pre- and peri-operative data.

	Estimated means (95% CI)	RM (IC 95%)	p-value
Male (n=309)	43269.11 (42175.87; 44390.70)	0.997 (0.961; 1.035)	0.888
Female (n=286)	43383.77 (42244.97; 44553.26)	1	
Age (years) (n=595)		1.001 (0.999; 1.002)	0.318
IMC (kg/m2) (n=575)		1.000 (0.995; 1.004)	0.927
Smoker (n=28)	39762.59 (36532.12; 43278.71)	0.914 (0.838; 0.997)	0.043
Non-smoker (n=567)	43500.11 (42688.67; 44326.97)	1	
Hypertensive (n=214)	42949.94 (41473.06; 44479.41)	0.987 (0.944; 1.031)	0.545
Not hypertensive (n=381)	43534.45 (42407.63; 44691.21)	1	
Diabetic (n=83)	43124.57 (41046.96; 45307.34)	0.995 (0.943; 1.049)	0.843
Non-diabetic (n=512)	43356.59 (42503.16; 44227.15)	1	
Heart disease (n=65)	41731.07 (39469.84; 44121.85)	0.959 (0.904; 1.017)	0.163
Non-cardiac (n=530)	43519.61 (42678.80; 44376.99)	1	
Surgery time (hours) (n=580)		0.995 (0.971; 1.020)	0.685
Length of stay (days) (n=585)		1.002 (0.994; 1.010)	0.586
1st PO in Infirmary (n=327)	43222.52 (42161.01; 44310.75)	1.003 (0.960; 1.047)	0.902
1st PO in Semi-ICU (n=108)	43957.63 (42096.25; 45901.31)	1.020 (0.964; 1.079)	0.493
1st PO in ICU (n=160)	43104.54 (41599.18; 44664.38)	1	

BMI, body mass index; MR, ratio of means; 95% CI, 95% confidence interval for the estimated ratio of means; reference categories have 1 in place of the odds ratio; PO, postoperative; ICU, intensive care unit

There was no evidence of a correlation between total cost and scores for quality of life (EQ-5D) and function (HOOS and WOMAC) at the end of two years (Table 6).

**Table 6:** Correlation coefficients of the total cost with the EQ-5D, HOOS and WOMAC scores at the end of two years.

		Total cost*
EQ-5D - 2 years	n=292	−0.079 (p=0.179)
HOOS score - 2 years	n=70	−0.041 (p=0.734)
WOMAC score - 2 years	n=223	0.093 (p=0.164)

* Spearman's correlation coefficient (p-value)

## DISCUSSION

In this study on THAs carried out in a private tertiary hospital with an open clinical body in Brazil, the most important results were to report an average total cost per patient of R$43,324.22, achieving satisfactory quality of life and functional results at one and two years of follow-up. Furthermore, there was no association between total cost and quality of life or functional outcomes. However, there was an association between the variation in EQ-5D two years after surgery and male gender and age.

The results of improved quality of life and function in patients undergoing THA were present at one and two-year follow-ups. At one-year follow-up, the EQ-5D showed an improvement of 0.396, which was greater than the 0.18 improvement one year after surgery in the study that analyzed more than 28,000 THAs in several European countries.^12^ This difference exceeds the *minimally important difference* of 0.074 for the EQ5D.^13^ There was also an improvement in the HOOS and WOMAC functional scores, similar to those found in other studies,^14–16^ reinforcing the excellent results of THA.

The total cost per patient at the end of hospitalization ranged from R$16,868.31 to R$147,082.01, with an average cost of R$43,324.22. The variation in cost is related to the different values of implants, materials inherent in the procedure and hospitalization costs. In an Italian public hospital, Fidanza et al^17^ cited an average value of €5,754.00 euros, relatively lower than the cost found in the present study, which was carried out in a private tertiary hospital. In the scenario of private American hospitals, Robinson et al^18^ and Gardezi et al^19^ reported a cost of between $2,391.00 and $12,651.00 dollars, comparable to the cost resulting from the present study. In England, from the national arthroplasty registry, between 2008 and 2017, around 400,000 THAs were registered, and an average cost of 6,208 pounds per patient was estimated,^20^ relatively lower than the average cost of this study.

The improvement in quality of life was associated with the preoperative factors age and gender. These associations have been found in other studies, but other factors such as previous surgeries, ASA, comorbidities and BMI have also been shown to be associated with clinical-functional outcome.^21,22^ This study found no association between these factors and quality of life, possibly due to the characteristics of the population and the number of patients included.

The increase in total cost has already been described as being associated with age, complication rate and fate after discharge,^18^ but in the present study there was no significant association with any preoperative factor, except smoking. Although patients who smoked had lower total costs than non-smokers, the interpretation of this finding is limited, as the frequency of smokers is small (N= 51; 4.8%), and there are no studies reporting this association. Furthermore, the lack of association between preoperative factors and total cost at an individual level, such as patient characteristics, seems to have a minimal influence on the final cost, with intra-hospital characteristics being most responsible for this variation, which could include materials and the particular preferences of each surgical team.^18^ This is corroborated by Fidanza et al,^17^ who cite that approximately half of the total cost is attributable to the value of the prosthesis alone. Given that the assessment of quality of life by predetermined scores limits the possible range of improvement and that hip arthroplasty is already recognized as a surgery of excellent effectiveness, regardless of the primary cause for surgery, it is not surprising that no associations were observed between total cost and quality of life or functional results up to 2 years after surgery.

This study has some limitations that should be taken into account. This is a retrospective study, which used an institutional database, without access to the medical records by the researchers, so that gaps in the data, sometimes incomplete, reduced the study's sample number for the different analyses carried out. However, the n used in each analysis is reported in the tables. Furthermore, as this was a single-center study in a single private tertiary hospital, its results cannot be extrapolated to the entire Brazilian population. However, it is a reference hospital in Latin America and open to external surgeons, whose profile, surgical technique and level of experience vary considerably.

## CONCLUSION

Patients who underwent THA in a private Brazilian hospital with an open clinical practice had improved quality of life and functional scores at one and two years’ follow-up and an average total cost per patient of R$43,324.22. In addition, there was an association between the increase in the EQ-5D score two years after surgery and male gender and age. There was no association between total cost and quality of life score.
